# Dynamic radiostereometric analysis for evaluation of hip joint pathomechanics

**DOI:** 10.1186/s40634-017-0096-2

**Published:** 2017-06-05

**Authors:** Lars Hansen, Sepp de Raedt, Peter Bo Jørgensen, Bjarne Mygind-Klavsen, Bart Kaptein, Maiken Stilling

**Affiliations:** 1 Department of Orthopedic Surgery, Aarhus University Hospital, Aarhus, Denmark; 2 Orthopedic Research Unit, Aarhus University Hospital, Tage-Hansens Gade 2, building 10A, Office 13, 8000 Aarhus, Denmark; 3 Department of Clinical Medicine, Aarhus University, Aarhus, Denmark; 4 NRT X-RAY, Hasselager, Denmark; 5 Department of Orthopaedics, Leiden University Medical Center, Leiden, the Netherlands

**Keywords:** Femoroacetabular impingement, Radiostereometric analysis, Biomechanics, Pathomechanics, Arthroscopy, Range of motion, Basic science, Hip

## Abstract

**Background:**

Dynamic RSA (dRSA) enables non-invasive 3D motion-tracking of bones and may be used to evaluate in-vivo hip joint kinematics including hip pathomechanics such as femoroacetabular impingement (FAI) and the biomechanical effects of arthroscopic cheilectomy and -rim trimming (ACH).

The study aim was to evaluate the kinematic changes in the hip joint after ACH.

**Methods:**

Seven non-FAI affected human cadaveric hips were CT-scanned and CT-bone models were created. dRSA recordings of the hip joints were acquired at five frames/s during passive flexion, adduction to stop, and internal rotation to stop (FADIR). ACH was performed and dRSA was repeated. dRSA images were analyzed using model-based RSA. Hip joint kinematics before and after ACH were compared pairwise. The volume of removed bone was quantified and compared to the postoperative range of motion (ROM).

**Results:**

Mean hip internal rotation increased from 19.1 to 21.9° (*p* = 0.04, Δ2.8°, SD 2.7) after ACH surgery. Mean adduction of 3.9° before and 2.7° after ACH surgery was unchanged (*p* = 0.48, Δ-1.2°; SD 4.3). Mean flexion angles during dRSA tests were 82.4° before and 80.8° after ACH surgery, which were similar (*p* = 0.18, Δ-1.6°, SD = 2.7). No correlation between volume of removed bone and ROM was observed.

**Conclusions:**

A small increase in internal rotation, but not in adduction, was observed after arthroscopic cheilectomy and -rim trimming in cadaver hips. The hip flexion angle of the FADIR test was reproducible. dRSA kinematic analysis is a new and clinically applicable method with good potential to evaluate hip joint kinematics and to test FAI pathomechanics and other surgical corrections of the hip.

## Background

Femoroacetabular impingement (FAI) is caused by an abnormality in the acetabular shape or orientation (Pincer-type), by a shape-abnormality in the proximal femur (Cam-type) or by a combination of the two (mixed-type) (Agricola et al. 2013; Beck et al. 2005). FAI most often presents in healthy, physically active, young persons (predominantly male) in the age range of 20–30 years (Ganz et al. 2003). It is recognized as a common cause of pain and early development of osteoarthritis (Larson et al. 2016; Kowalczuk et al. 2015). The reported prevalence of asymptomatic FAI in radiographs is 23–32% for CAM lesions and 43–67% for pincer lesions (Frank et al. 2015; Diesel et al. 2015). Studies show that physical impairments for individuals with symptomatic FAI primarily consist of motions bringing the hip towards impingement. Typically impaired daily activities are stairclimbing, squat and restrictions in frontal, transverse and sagittal hip motion during gait (Diamond et al. 2015a; Diamond et al. 2015b). Further, studies have shown that FAI patients lack hip muscle strength compared to normal controls (Casartelli et al. 2011; Freke et al. 2016).

The preferred surgical treatment of FAI is by arthroscopic cheilectomy and -rim-trimming (ACH) (Nwachukwu et al. 2015). Excess bone is removed in the head-neck transition of the femur bone and in the anterolateral region of the acetabular rim. Arthroscopic technique is superior to an open approach based on higher postoperative general health-related quality of life (HRQoL) score (Nwachukwu et al. 2015) and an increased patient satisfaction of 82% (Sansone et al. 2016). Still the main reason for revision after ACH procedure is failure to identify and/or reshape the affected areas in the joint adequately (Heyworth et al. 2007; Ross et al. 2014; Philippon et al. 2007; Larson et al. 2014).

Earlier studies have investigated joint kinematics related to FAI pre- and postoperatively. Simulation studies using CT-reconstructed bone models for simulation of impingement positions have been performed (Bedi et al. 2011; Kubiak-Langer et al. 2007; Sampson & Safran 2015). Limitations of this method are that it commonly assumes range of motion (ROM) to be governed by bone-bone contact, and they do not track the exact in vivo motions of the bone (Kapron et al. 2015). Advantages of simulation studies are that no large setup is required and patients are only exposed to radiation in relation to the CT-scan that is used to create bone models. Motion capture systems primarily investigate functional in-vivo hip kinematics during gait or squat, but do not investigate ROM during passive movements (Diamond et al. 2015a; Brisson et al. 2013; Rylander et al. 2011), subluxation of the hip joint (translation of the femur center of rotation with respect to the femur) and bone-bone distances due to soft tissue artifacts (Taylor et al. 2005). Kapron et al. used dual fluoroscopy and a digitally reconstructed radiograph based analysis method for tracking bone movements during flexion, adduction and internal rotation (FADIR), and investigated in vivo kinematics of the hip joint in three FAI-patients and six non-FAI participants (Kapron et al. 2015; Kapron et al. 2014). They found that the FAI-group had decreased adduction and internal rotation during passive tests and further that ROM is governed by labrum contact and other soft tissue restraints in the native joint. They did not investigate post-operative changes in kinematics.

The pathomechanics for development of symptoms in FAI are not well understood, and neither are the kinematic changes in relation to arthroscopic surgery. The establishment of methods for understanding the objective kinematic changes in the hip joint following ACH will provide evidence of the efficacy of surgery (Bedi et al. 2011).

The aim of this study was to establish methods for evaluation of hip-joint kinematics before and after ACH. Human cadaveric specimens without FAI were used in the experiments. We hypothesized that increased hip ROM (rotations) would be measureable with model-based dynamic RSA (mbRSA) after ACH surgery.

## Methods

### Specimens

Seven human cadaveric legs including hip joints and hemipelvises, from 4 donors were used in the study (Department of Biomedicine, Aarhus University). The age of the donors ranged between 58 and 94 years, three were from male- and four from female donors. Inclusion criterion was no prior hip surgery, which was assessed by x-ray of the hip and visual inspection for earlier surgical incisions. The study was approved by The Central Denmark Region Committees on Health Research Ethics (Case number 1-10-72-6-16 issued on February 24th, 2016).

The donor legs were scanned in a computed tomography (CT) scanner (Brilliance 64, Philips Healthcare, Cleveland, OH, USA). Settings were 120 kV, 150 mAs, slice thickness 2.5 mm and slice increment 1.25 mm. Bone models were created using an automatic graph-cut segmentation method (Krčah et al. 2011; de Raedt et al. 2013). Bone segmentations of the pelvis included the iliac-, ischial- and pubic bone and for the femur the head down to 5–7 cm distally to the lesser trochanter. All segmentations were visually inspected and verified to be within voxel accuracy (<0.3 mm). Local coordinate systems were created for the bone models by the method described by Wu et al. (Wu et al. 2002).

### Experimental setup and equipment

**Fig. 1:**
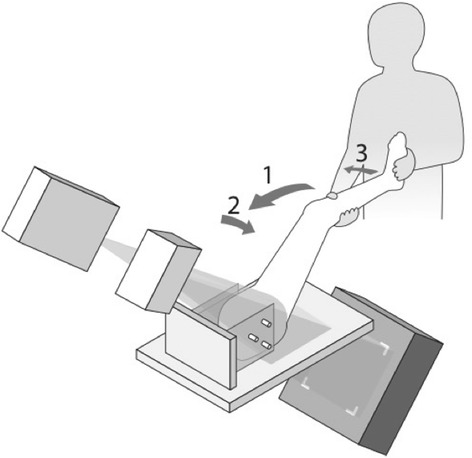
Setup of the radiostereometric equipment. The x-ray tubes were positioned with 20° medio-lateral and 45° cranio-caudal tilt. The calibration box was placed in a 45° angle beneath the hip joint. The FADIR motion is indicated by the numbered arrows: 1) Flexion to 90° 2) adduction to stop 3) internal rotation to stop

**Fig. 2:**
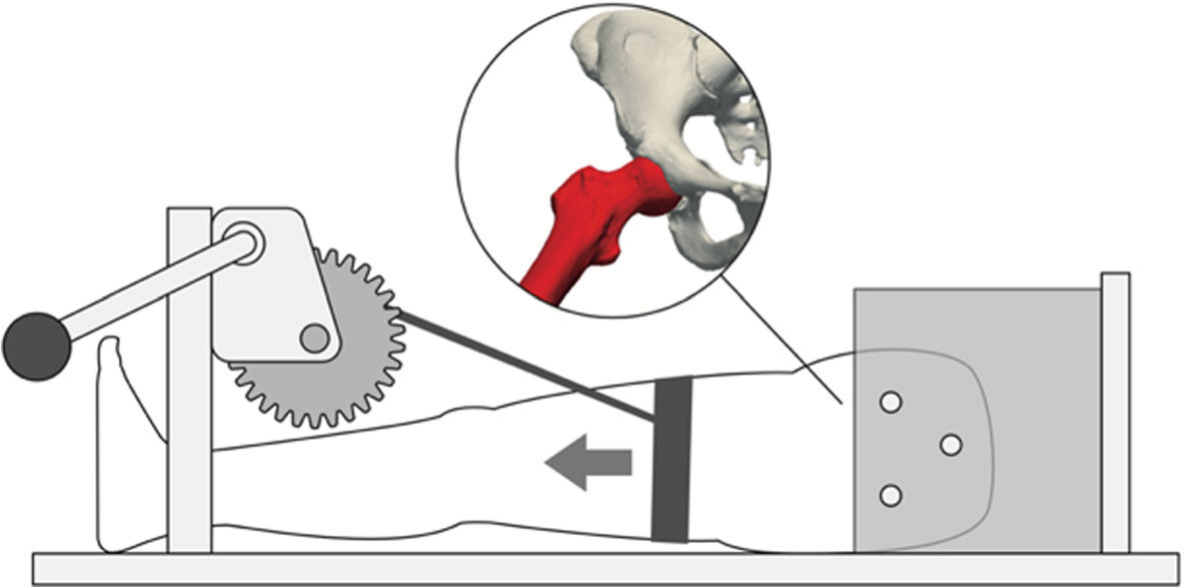
The surgical setup. The pelvis was mounted in a portable fixture using three spiral drills. Traction was applied using a winch which could be adjusted in height to change flexion angles during ACH

**Fig. 3:**
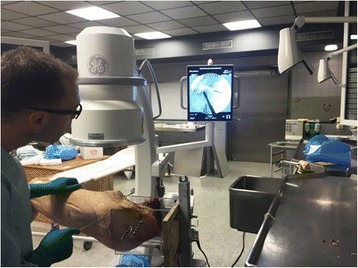
Images of the surgical setup during ACH. The lateral portal was placed using fluoroscopic guidance

**Fig. 4:**
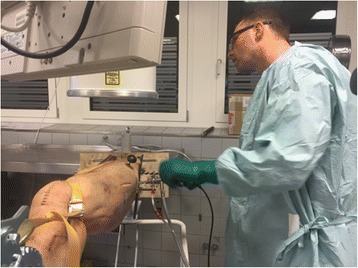
Images of the surgical setup during ACH. The lateral portal was placed using fluoroscopic guidance

A portable fixation for the hemipelvises which could be mounted both to the radiology table during recordings and in an operative setting during ACH was constructed (Figs. 1, 2, 3 and 4). Fluoroscopy was made possible from the medial side and used for entering the joint and evaluating the amount of traction applied. Traction was applied using a winch by pulling on a strap around the distal femur. ACH was performed with a 70° wide angle arthroscope, a radiofrequency wand (super multivac 50), burr (5.5 mm barrel burr) and a shaver (dyonics incisor plus), (all surgical equipment was provided by Smith and Nephew, London, United Kingdom). Resection was performed by an experienced arthroscopist with simulation of usual FAI-surgery, allthough the donor hips were not FAI hips by radiological definitions. The surgeon restored what appeared to be normal morphology in accordance with the Danish Hip Arthroscopy Registry (DHAP) (average circumferential area of 116° (SD = 24.5) and a mean depth of 3.8 mm (SD = 1.7) (Lund et al. 2017).

### Radiographic setup

All stereoradiographs were recorded using a dynamic RSA system (Adora RSAd, NRT X-Ray, Denmark). Sampling frequency was five frames/s, pulse width 16 ms. Roentgen tubes were positioned with a 45° cranio-caudal- and 20° medio-lateral tilt directed at the hip joint from the cranial-caudal direction. Beneath the table a uniplanar calibration box (Box 14; Medis Specials, Leiden, the Netherlands) was placed in a 45° angle to the horizontal plane (Fig. 1). The two image detectors (Canon CXDI-50RF) were slotted in the calibration box. Source image distance (SID) was 2220 mm and focus skin distance (FSD) 1140 mm. Exposure settings for dRSA recordings were 130 kV, 500 mA, 16 ms and resolution was 1104x1344 pixels (79 DPI).

### Test protocol

Preoperatively the cadaver specimens were CT-scanned and dRSA was performed. One dynamic RSA recording of the hip during FADIR motion, which is the movement of the donor leg from full extension through flexion aiming at 90°, adduction to stop, and internal rotation to stop (end range) was made (Fig. 1). The FADIR motion was performed slowly due to low frame rate and to ensure a controlled motion. ACH was performed by the senior surgeon (BMK). Postoperatively dRSA was repeated and a postoperative CT-scan of each specimen was performed.

### Analysis of radiographs

For analysis of radiographs the commercially available software model-based RSA 4.01 (RSAcore, LUMC, Leiden, The Netherlands) was used. For each specimen calibration of the image was performed in the first frame. For the mbRSA-analysis the created bone models were implemented in the software program. Contours of the pelvic- and femur bones were detected on the two simultaneous images of the same scene by the Canny Edge Detector and relevant contours were manually identified, aiming to use similar contours in each frame (Kaptein et al. 2004). The software automatically positions the bone models using three consecutive algorithms: IIPM, DIFDHSAnn and DIFDoNLP. These algorithms estimate the pose by minimizing error between the virtual projections of the bone models and the manually detected contours on the radiographs (Kaptein et al. 2004). For each specimen the frame in the sequence, in which the hip was in end range FADIR, was identified and used to determine flexion, adduction and rotation angles of the hip joint (calculated according to the ISB recommendation (Wu et al. 2002) along with femoral end-range subluxation (the norm (T^2^ = *X* ^2^ + Y^2^ + Z^2^) of translations of the femur bones’ center of rotation with respect to the pelvis). The translation was the difference in position of the center of the femur between two frames in the femur coordinate system.

The CT-scans of the separated hemipelvises were aligned with the contralateral side to determine the anatomic coordinate system and subsequently the lateral center-edge angle (CE) and alpha angle were calculated to determine the preoperative degree of FAI by Clinical Graphics (Delft, The Netherlands).

**Fig. 5:**
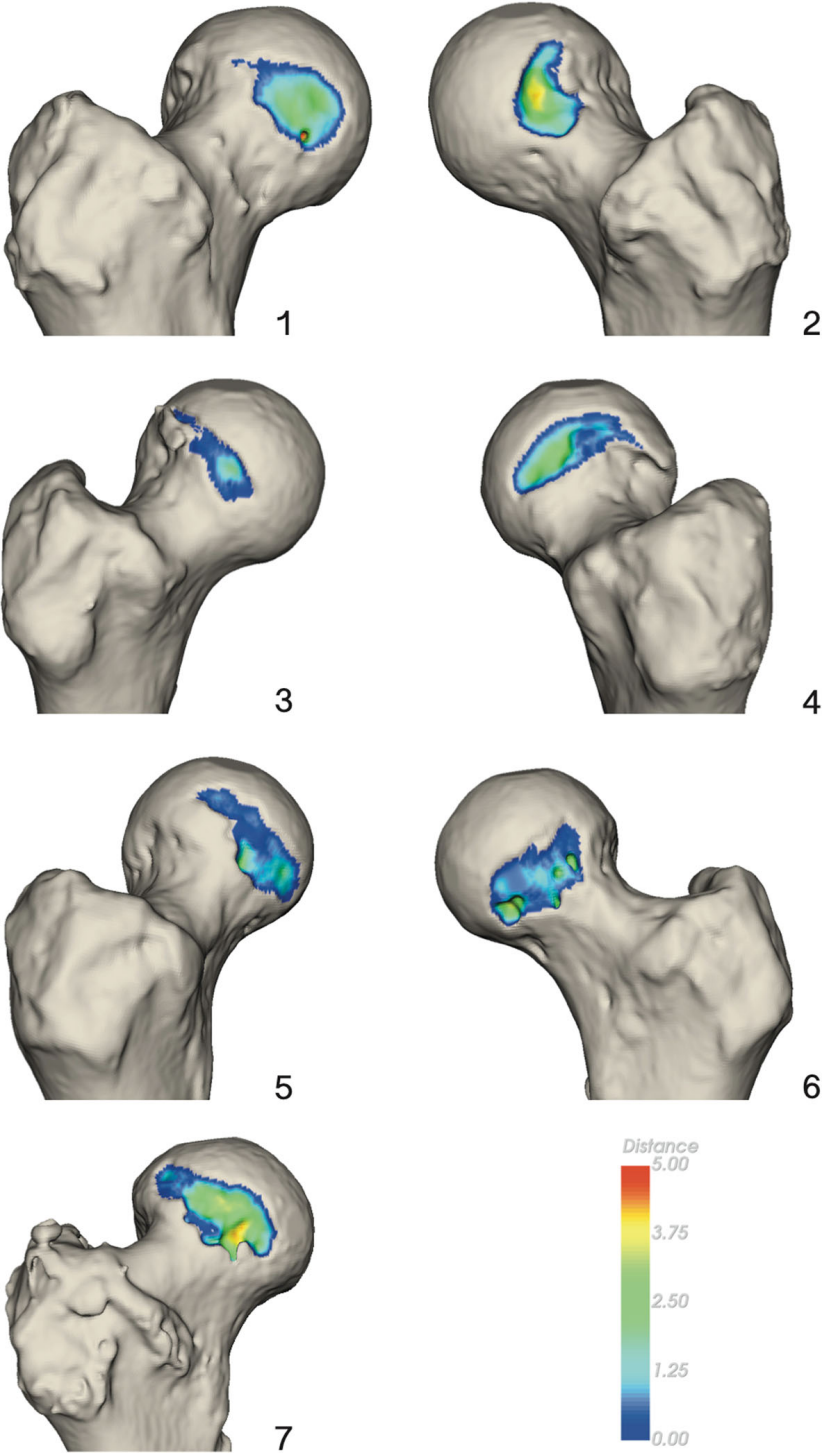
Figure showing the area and depth of resected bone during ACH for all seven donor hips. The color scale refers to depth of resection in millimeters

The volume of removed bone after ACH was determined by aligning the pre- and post-operative CT scans using image registration and segmenting the region with an intensity change above 50 Hounsfield units (Klein et al. 2010). The resulting model represents the post-operative bone showing the area where bone was removed during ACH. The depth of the removed bone was calculated as the distance from each point of the post-operative surface to the closest point on the pre-operative surface (Fig. 5).

### Radiation dose

Based on real time dRSA recordings dose-calculations were performed. The revealed effective dose per exposure was 0.054 mSv. Recordings were acquired at 5 frames/s with a mean exposure time of 9 s giving an effective dose of 2.43 mSv per recording. The CT-scan contributed with an effective dose of 10 mSv per scan. Total effective dose was 24.86 mSv per specimen.

### Data analysis

Data was summarized as flexion-, adduction- and internal rotation angles as measures of ROM. Paired t-tests were used to compare pre- and postoperative results. Scatterplots of the volume of removed bone against flexion, adduction and internal rotation respectively were constructed to check for correlations. End range sub-luxation was measured as the norm of translations along the x-, y- and z-axes by use of the 3D Pythagorean theorem (T^2^ = *X* ^2^ + Y^2^ + Z^2^). Pre- and postoperative sub-luxation was compared using paired t-tests. The statistical significance level was set to 5% and Stata/IC 14.1 (StataCorp, College Station, Texas, USA) was used for statistical analyses.

### Precision of mbRSA

In an earlier validation study, we have validated dynamic mbRSA as a method for evaluation of hip kinematics in FAI (unpublished work). Validation was performed using traditional marker based RSA as gold standard. Precision of mbRSA described as 95% limits of agreement (± LOA) (standard deviation * 1.96) were below 0.44 mm for translations of the femur, below 0.91 mm for translations of the pelvis and below 0.7° in rotations for both the femur and pelvis.

## Results

**Fig. 6:**
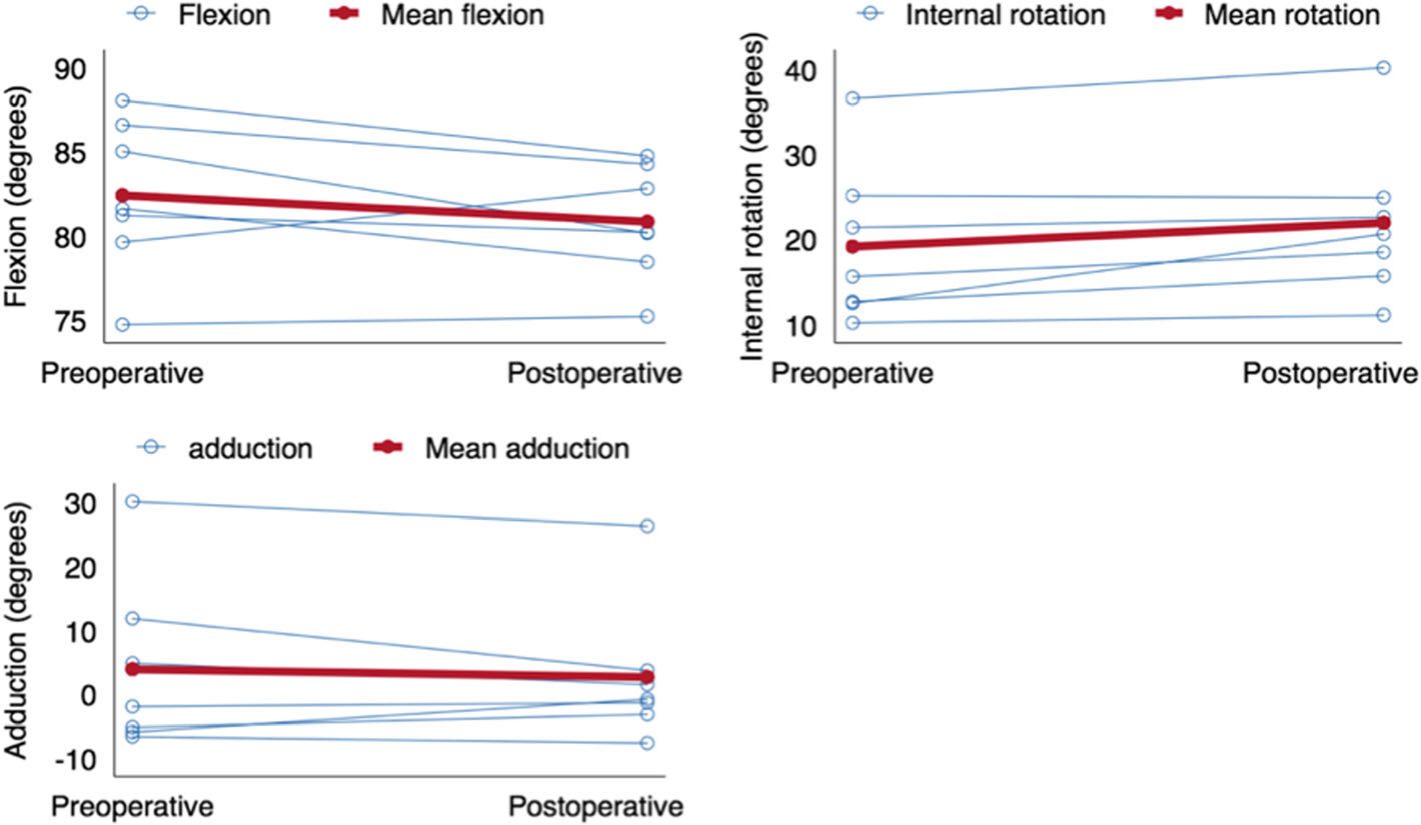
Scatter plot showing the development in flexion, internal rotation and adduction between the pre- and postoperative investigation

**Fig. 7:**
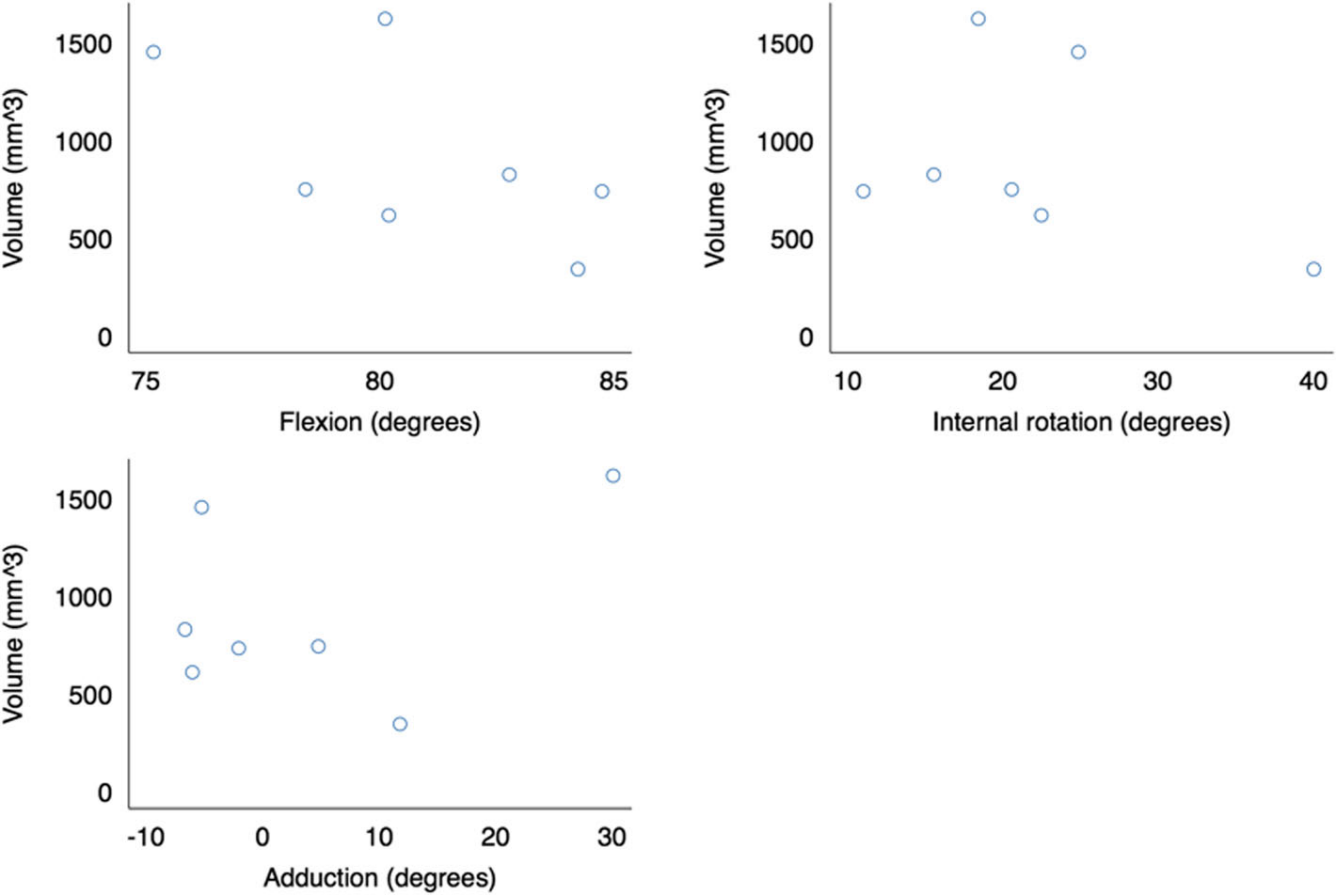
Scatter plots of the volume of removed bone with respect to the postoperative flexion, internal rotation and adduction

The kinematic results showed a postoperative mean increase in hip rotation of 2.79° (SD 2.7; *p* = 0.04). No increase in adduction was observed (mean difference 1.23°, SD 4.3; *p* = 0.48) and no statistical difference in flexion was found between pre- and postoperative recordings, mean difference −1.57° (SD 2.7; *p* = 0.18) (Fig. 6). Mean pre- and postoperative flexion angles were 80.8 and 82.4° respectively. The flexion angles for the individual donors varied between 75 and 87° but no significant development from pre- to postoperative was observed (Fig. 6). No correlation was found between ROM and volume of removed bone (Fig. 7).

A large variation in the volume of removed bone on the femur was observed with a mean volume of 894 mm^3^ (SD 459 mm^2^) and minimum and maximum values of 335 and 1609 mm^2^ (Table 1).

**Table 1:** Table showing the preoperative CE- and alpha angle and volumes of removed bone during ACH for the seven donor hips

Donor ID	CE (°)	Alpha (°)	Volume (mm^3^)
1	39.5	43.0	816
2	43.1	48.3	734
3	39.2	43.4	335
4	47.4	43.2	1609
5	36.8	48.1	604
6	36.7	42.3	724
7	28.1	51.6	1439

Mean pre- and postoperative subluxation at end range FADIR, 3.9 and 3.5 mm respectively, did not differ significantly (SD 0.96; *p* = 0.37). Also no differences were observed (*p* > 0.05) when comparing the translations for the individual degrees of freedom.

Measurements of CE and alpha-angles revealed that none of the donor hips had a cam-lesion (α < 55°) but showed that two of the donors had a CE > 40° and thereby per definition a pincer-lesion (Table 1).

## Discussion

Dynamic radiostereometry was used to investigate kinematic angles in the hip joint of human cadaver specimens during a passive FADIR motion before and after ACH, and the key finding was a small statistically significant increase of 2.79° in internal rotation but no increase in adduction. The mean removed bone was 894 mm^3^.

The use of cadaver specimens make up the best imitation of a clinical setting and we have shown mbRSA evaluation of hip kinematics to be very precise (see “Precision of mbRSA”). However, there are a number of limitations in this study related to the use of cadavers. After ACH the joint space was meticulously emptied for excess water to reduce the influence on measurements. However, the increase in internal rotation was smaller than expected and might be explained by water accumulation in the tissue around the hip causing edema and rigidity. No difference in pre-and postoperative hip joint subluxation was observed, and therefore eventual loss of muscle tone stabilization after traction on the hip joint and eventual postoperative capsule laxity after distension during arthroscopy cannot explain the small post-operative increase in hip ROM. Since flexion angles did not differ significantly, they too do not explain the low increase in internal rotation. In patients, we would expect blood circulation and recovery time after surgery before control measurements would be possible to eliminate this limitation and provide greater kinematic improvements after ACH.

Due to the high age of specimens the bone quality was low and labrums were calcified, which made it more difficult to determine the border between the labrum and the acetabular bone on the CT-scans. Hereby, much of the labrum was segmented along with the bone during model-construction making the pelvis bone model less accurate in the acetabular rim region. Therefore, measurements of the CE angle are expected to be higher and it was not possible to measure the amount of bone removed from the acetabulum. Further, the inability to differentiate between the bone and the labrum made it impossible to measure bone-bone distances at impingement and determine whether the bones collided at end range FADIR. Further, the low bone quality influenced the conditions for bone removal at a consistent depth because the burr would easily penetrate into the bone in soft regions. This may have contributed to the large variation between subjects in volume of removed bone.

The cadaver fixture and fixation had to allow for stereoradiography and therefore only a small area of the ilium could be used to ensure that the fixation did not block the x-rays. At end range FADIR the mean pre- and postoperative hip flexion angles were measured to be 80.8 and 82.4° with RSA, while we anticipated to reach 90° clinical flexion during testing. This may also be attributed to the use of cadaveric hemipelvises which made it more difficult to estimate the exact flexion angle during the experiment. Yet, due to the variation in pelvic tilt and the variation between patients this may also be a challenge in clinical studies. However, reproducibility to reach the same flexion position pre- and post-operative was good with a mean difference of −1.57°.

The CT-scans and RSA examinations contributed with a combined effective dose of 24.86 mSv. During the study further tests have been performed on the required quality of the CT-scans. A new CT-scanner has been installed at our institution and the field of view has been decreased to include only the joint space for the postoperative scan. This will allow for a substantial dose reduction of the CT-scans to 5.2 mSv for the preoperative scan and further reduction for the postoperative scan, which is necessary for estimating the volume of resected bone. In addition further reductions in the effective dose of the RSA examinations will and can be achieved. The reduction in radiation dose will justify the use of mbRSA for future clinical use in FAI patients, when taking the severity and prevalence of FAI into account (Radiation protection 99, EU guidelines). Furthermore, the kinematics can be determined without the post-operative CT scan by using the models created from the pre-operative CT scan, which would further reduce the dose in clinical use. However, then no estimate of the removed bone can be calculated.

To our knowledge only one very small numbered in-vivo RSA study evaluating hip joint kinematics has formerly been conducted. Kapron et al. used a digitally reconstructed radiograph based method for preoperative in vivo kinematic investigations of the hip on six normal subjects and three symptomatic FAI subjects. They suggested that the restriction of hip ROM is governed by the labrum and other soft tissue constraints (Kapron et al. 2015). Since only three symptomatic FAI patients were included, no statistical comparison was performed. For the asymptomatic group Kapron et al. found a mean internal rotation of 19° and a mean adduction of 11°. In comparison, we found a similar mean preoperative internal rotation of 19.1° but a lower mean adduction of 3.9°. The mean adduction found in this study was comparable to the results of the symptomatic group found by Kapron et al. Roach et al. have investigated digital inclinometer and goniometer for measuring passive hip motion and found these methods to be associated with errors up to 5° (Roach et al. 2013). Hence, these methods would not be feasible for measuring the differences in ROM found in this study nor for measuring the differences between subjects (Kapron et al. 2015).

The pain reduction after ACH that has been reported in patients might not be caused by improved adduction and internal rotation but by a reduction in labral stress in the resected regions (Nwachukwu et al. 2015; Sansone et al. 2016). Applying mbRSA for evaluation of FAI hips in a clinical study could provide further insight of the in-vivo pathomechanics of FAI and the mechanisms causing pain. mbRSA has proven to be an applicable tool for in-vivo bone tracking and has potential to be used for evaluation of other corrective interventions of the hip such as periacetabular osteotomy in hip dysplasia (Liu et al. 2015; Murphy et al. 2016). A better understanding of the biomechanics relating to various hip conditions may improve the understanding of the etiology and thereby improvements in treatment and surgical correction.

In this study we have shown that hip internal rotation increases after ACH in cadaver hips, that flexion angles during a passive FADIR test may be reproduced, and that the volume of removed bone on the femur can be quantified. Importantly, the study has provided valuable knowledge concerning the RSA set-up, exposure settings, CT-protocol, patient-positioning and other details needed in order to apply dynamic RSA with bone-models in clinical use for evaluation of hip kinematics. In the future, this method may provide surgeons with the necessary insight to further improve patient outcome and satisfaction when using ACH.

## Abbreviations

ACH: Arthroscopic cheilectomy and -rim trimming
CE: Center-edge angle
dRSA: Dynamic RSA
FADIR: Flexion, adduction and internal rotation
FAI: Femoroacetabular impingement
FSD: Focus skin distance
HRQoL: General health-related quality of life
mbRSA: Model-based radiostereometric analysis
ROM: Range of motion
SID: Source image distance
